# The extracellular matrix microtopography drives critical changes in cellular motility and Rho A activity in colon cancer cells

**DOI:** 10.1186/1475-2867-10-24

**Published:** 2010-07-28

**Authors:** Rebecca Rapier, Jameela Huq, Ramana Vishnubhotla, Marinka Bulic, Cecile M Perrault, Vitali Metlushko, Michael Cho, Roger Tran Son Tay, Sarah C Glover

**Affiliations:** 1 University of Illinois at Chicago, Chicago, IL USA; 2 Jesse Brown VA Medical Center, Chicago, IL USA; 3 University of Florida at Gainesville, Gainesville, Fl USA

## Abstract

We have shown that the microtopography (mT) underlying colon cancer changes as a tumor de-differentiates. We distinguish the well-differentiated mT based on the increasing number of "pits" and poorly differentiated mT on the basis of increasing number of "posts." We investigated Rho A as a mechanosensing protein using mT features derived from those observed in the ECM of colon cancer. We evaluated Rho A activity in less-tumorogenic (Caco-2 E) and more tumorigenic (SW620) colon cancer cell-lines on microfabricated pits and posts at 2.5 μm diameter and 200 nm depth/height. In Caco-2 E cells, we observed a decrease in Rho A activity as well as in the ratio of G/F actin on surfaces with either pits or posts but despite this low activity, knockdown of Rho A led to a significant decrease in confined motility suggesting that while Rho A activity is reduced on these surfaces it still plays an important role in controlling cellular response to barriers. In SW620 cells, we observed that Rho A activity was greatest in cells plated on a post microtopography which led to increased cell motility, and an increase in actin cytoskeletal turnover.

## Background

Mechanical signals sent to cells through physical changes in the tumor microenvironment are becoming increasingly understood as important regulators of tumor cell behavior. There is a long history of evaluating the contribution of the topographical features of surfaces on cell behavior in the bioengineering literature. In studies performed to date, the topographical features of a surface have been shown to affect cell adhesion [1-8], migration [2,9], motility [1], survival [10,4], growth [3,11,7], and differentiation [12], with different cell types responding differently to specified textures. These studies have almost uniformly focused on how to bioengineer artificial materials so as to more readily ensure their acceptance *in vivo*, and as such, have been performed in the context of improving implant and graft survival. In contrast, few studies have been performed evaluating the effect of surface texture, either synthetic or that which has been natively synthesized, on the behavior of malignant cells.

Cells change their behavior through the traction forces they exert via their actin cytoskeleton on their surrounding extracellular matrix (ECM) [13]. Investigators studying cell behavior within the pulmonary, cardiovascular, and musculoskeletal system have long understood the importance of mechanical signals to cell behavior and have identified numerous mechanosensing proteins, including the Rho family of proteins. Rho has been shown to regulate traction forces [14,15], to be a critical regulator of intracellular responses to micromechanical properties of the ECM [16-18], to aid in cellular response to physical surroundings in a spatially oriented manner [19], and to be an important regulator of integrins in mechanical tasks of high complexity in three-dimensional surroundings [20]. In addition, these proteins have been linked with epithelial differentiation in response to flexible surroundings [21] as well as to stem cell commitment [22,23].

In cancer, Rho has been linked with tumor cell invasion. In esophageal cancer, shear stress has been shown to increase tumor cell invasion, which is negated by the presence of a ROCK inhibitor [24]. In breast cancer, ERK and Rho have been shown to constitute part of an integrated mechanoregulatory circuit linking matrix stiffness to cytoskeletal tension through integrins to regulate tissue phenotype [25] This mechanical "autocrine loop" brings "solid-state mechanotransduction on a par with oncogenic signaling pathways in malignant transformation [26,27]."

There are two ways to classify tumors: well-differentiated tumors and poorly differentiated tumors. Well-differentiated tumors describe tumors which have some resemblance to its original tissue. Poorly differentiated tumors represent tumors which have little resemblance to the original tissue. These cells have properties that resemble stem cells. The process by which cells change phenotype from epithelial cells to stem cells is known as de-differentiation. Poorly differentiated cells, such as SW620 cells, are more likely to be metastatic with respect to well-differentiated cells, such as Caco-2 E cells.

In our previous research, we have shown that the microtopography underlying colon cancer cells changes as the tumor de-differentiates [28]. Specifically, we demonstrated that the matrix underlying well-differentiated tumor cells has more "pits" while the matrix underlying poorly differentiated tumor cells has more "posts". Additionally, we have shown that changes in the ECM microtopography affect intracellular signaling processes [29]. Based on our data as well as others data, we hypothesized that the microtopography of the ECM has the ability to alter colon cancer cell motility via modulation of Rho A signaling.

To test this hypothesis, we created micron scale topographies in Polydimethylsiloxane (PDMS), a silicon based organic polymer, and evaluated the behavior of less tumorogenic, Caco-2 E cells and tumorogenic, SW620 cells. An ELISA based assay was used to evaluate Rho A activity. Time-lapse photography and a G/F actin ratio were used to understand the impact of the microtopography on cell motility and on actin turnover. Finally, to evaluate the contribution of Rho A to motility on these surfaces was evaluated using siRNA targeted against Rho A.

## Materials and methods

### Reagents and Supplies

DMEM/F-12 and Leibovitz's L-15 were purchased from Mediatech, Inc. (Herndon, VA) and Opti-MEM was purchased from Invitrogen (Carlsbad, CA). Fetal bovine serum (FBS) was purchased from Gemini Bio-Products (Sacramento, CA). Caco-2 E cells were obtained from Jerold Turner, MD, Ph.D. at the University of Chicago. Cells were maintained in dishware from BD Falcon (Lincoln Park, NJ). Lysis buffer was treated with Protease inhibitor cocktail from Sigma-Aldrich (St. Louis, MO). Cell extracts were equalized using the BCA Protein Assay Kit from Pierce (Rockford, IL). All immunocytochemical supplies, with exception of the antibodies, were from DAKO Cytomation (Carpentaria, CA). Antibodies recognizing Rho and beta-actin as well as HRP conjugated secondary antibodies were from Santa Cruz (Camarillo, CA). Florescent staining was done with FITC labeled anti-goat IgG from Sigma-Aldrich. All other florescent stains were obtained from Invitrogen. Western blot analysis was performed using the ECL Plus detection system from Amersham (Piscataway, NJ). Rho A activity was found using the "G-LISA Rho A activation assay biochem kit" available through Cytoskeleton (Denver, CO). For Actin Experiments, "G-actin/F-actin in Vivo Assay Kit" and Pyrene Labeled Muscle Actin were both acquired through Cytoskeleton (Denver, CO). siRNA experiments were performed with siRNA against Rho and siCONTROL from Dharmacon (Lafayette, CO). Other siRNA reagents, such as transfection reagent and medium, were from Santa-Cruz (Santa Cruz, CA). HMDS (hexamethyldisilazane) and other electron microscopy supplies were purchased form Electron Microscopy Sciences (Hatfield, PA). Sylgard 184 PDMS was purchased from Dow Corning (Midland, MI). All other supplies were molecular biology grade and were from Fisher (Pittsburg, PA).

### Creation of Scaffolds

**Figure 1:**
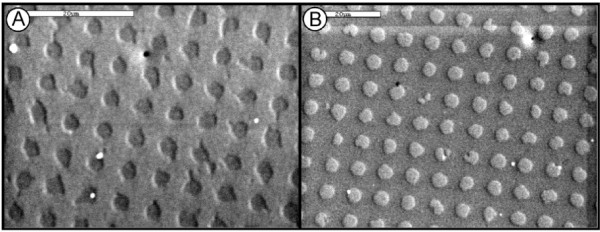
**SEM images of PDMS scaffolds used to re-create the ECM microtopography**. The Pit microtopography emulates the ECM under well-differentiated tumor cells (A). The Post microtopography emulates the ECM under poorly differentiated tumor cells (B). The pitch of each scaffold is 6 μm. The depth of the pits and height of the posts is 200 nm. Planar PDMS (not shown) was used as a control. Bar = 20 μm.

Masks were made using standard lithography techniques in the Nanotechnology Core Facility at the University of Illinois at Chicago. Pit and post masks were made to have a pitch of 6 um with depth or height of 200 nm (Figure 1). The control scaffold was created using a mask with a planar surface. To make the PDMS microtopographies, a silicon elastomer with curing agent was poured on the mask. These scaffolds were allowed to degas at room temperature for 30 min, and set for 3 hr at 75°C. PDMS scaffolds were then removed from the mask, sized to the required culture plate and rinsed in 100% Et-OH. The scaffolds were then placed into the culture plate pattern side up so that the pattern would contact the cells. The plates were then irradiated in a cell culture hood under UV overnight to decontaminate them prior to use in tissue culture experiments.

### Cell Culture

Caco-2 E cells, a subclone of Caco-2 BBE developed in the laboratory of Dr. Jerold Turner at the University of Chicago, were cultured in DMEM/F-12 50/50 1× with L-glutamine and 15 mM HEPES supplemented with 10% fetal bovine serum and were incubated at 37°C in a 5% CO_2 _atmosphere. SW620 cells were cultured in Leibovitz's L-15 medium 1× with L-glutamine supplemented with 10% fetal bovine serum and were incubated at 37°C in a 1% CO_2 _atmosphere.

### Seeding of Cells

**Figure 2:**
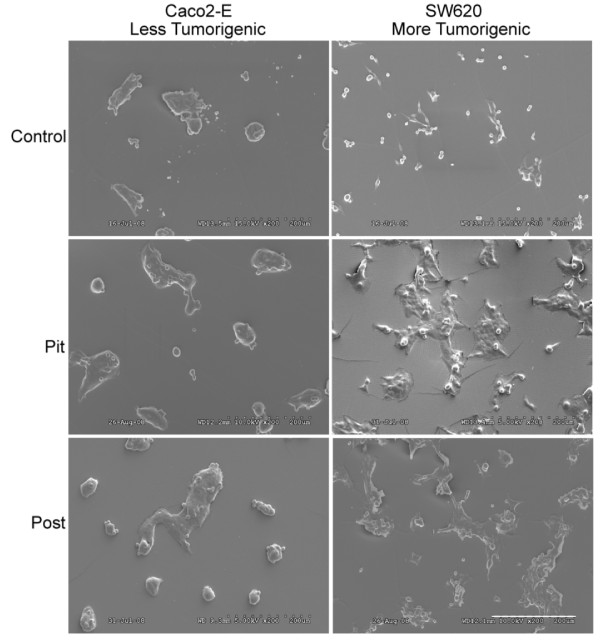
**Colon cancer cells adhere to PDMS scaffolds**. Cells were seeded on pre-treated PDMS scaffolds and cell containing scaffolds were prepared for scanning electron microscopy as described in the methods section of this paper. Cells adhered to each microtopography studied. Bar = 200 μm.

PDMS microtopographies were fitted into 100 mm plates and pre-incubated with Opti-MEM overnight at 37°C. Small amount of serum adsorbed to the surface, aiding cell adhesion. Opti-MEM was removed and cells were seeded at a density of 150,000 cells per cm^2^. Cell containing microtopographies were returned to the incubator for 30 minutes prior to the addition of Opti-MEM to allow them to adhere. Cell containing scaffolds were fixed using Trump's fixative and dehydrated in graded ethanol followed by HMDS. Cell containing scaffolds were then sputter coated with 5 nm of platinum palladium using a Cressington sputter coater. Scanning electron micrographs were then obtained using a Hitachi S-3000N to verify adherence to the various topographies (Figure 2).

### Rho A activity

Seeding of cells was preformed as described above. Rho A activity was evaluated at 24 hours after seeding using a colorimetric Rho A activity assay from Cytoskeleton, Inc. The assay was performed according to the manufacturer's instructions. Briefly, cells were lysed using an ice cold proprietary lysis buffer followed by clarification by centrifugation at 10,000 rpm at 4°C for 2 min. The supernatant was then collected and the protein concentration was equalized using the components provided by the kit. Using the kit provided 96-well plate, 25 μL of equalized cell extract was bound to the wells using binding buffer and placed on an orbital shaker at 400 rpm at 4°C for 30 min. The plate was subsequently incubated at room temperature in Antigen Presenting Buffer for 2 min, anti-Rho A primary antibody for 45 min, secondary antibody for 45 min, and HRP detection reagent for 15 min, with washes between each incubation period. The absorbance of each sample was measured in triplicate at 490 nm and recorded using a microplate spectrophotometer (Model 680XR, BioRad). Each sample reading was normalized by subtracting the reading from the blank from the sample reading. The Rho A activity for each condition was then determined by taking an average of the normalized sample readings over multiple experimental sessions (n = 4).

### Interference with Rho A protein expression

Cells were plated onto 35 mm plates at a density of 1.5 × 10^6 ^cells per plate and incubated in the appropriate cell type media. After 24 hrs, the plates were transfected with either 20 nmol siRNA against Rho A or 20 nmol of a scrambled siRNA sequence. The cells were then incubated in serum-free, antibiotic-free transfection medium for 6 hrs. The cells were then incubated in standard media with 20% FBS for an additional 48 hrs and were used within 72 hrs of their initial transfection.

To confirm effective knockdown of Rho A, cells were lysed using primary lysis buffer (50 mM HEPES, 150 mM sodium chloride, 1.5 mM magnesium chloride, 100 mM sodium fluoride, 10 mM sodium pyrophosphate) containing 1:50 dilution of protease inhibitor cocktail. Protein concentrations were determined using the BCA reagent kit with 40 *μ*g of each extract electrophoresed per lane on a 15% polyacrylamide gel under denaturing and reducing conditions. The resolved proteins were electrophoretically transferred to PVDF membranes. Immunoreactive bands were visualized using Rho A at 2 μg/mL. Appropriate loading was confirmed using beta-actin at 2 μg/mL followed by horseradish peroxidase conjugated goat anti-mouse IgG and the ECL Plus detection system.

### Time Lapse Photography and Analysis of Motility

PDMS microtopographies were fitted into 35 mm plates. Untransfected and transfected cells were seeded at a density of 150,000 cells per cm^2^. The cells were allowed to attach over a 15 to 20 minute period at 37°C prior to the addition of Opti-MEM without additional serum or antibiotics. Each 35 mm plate was then placed in a 37°C incubation chamber (MI-3, Precision Assemblies Co.) and visualized using a 40× objective over a 5 hour period. During that period, images were acquired every 5 minutes using an Olympus Microfire camera. Cell positions were tracked in consecutive video frames using the Manual Tracking plug-in (MTrackJ) for ImageJ (National Institutes of Health, Bethesda, MD, U.S.A.).

### G/F Actin

Cells were plated as described above. Cells were lysed using collected using a buffer containing 50 mM PIPES, pH 6.9, 50 mM potassium chloride, 5 mM magnesium chloride, 5 mM EGTA, 5% (v/v) glycerol, 0.1% NP-40, 0.1% Triton X-100, 0.1% Tween-20, 0.1% 2-mercaptoethanol, and 0.001% AntifoamC. The lysates were then centrifuged at 2000 rpm for 5 min. The supernatant was collected and subsequently centrifuged at 100,000 × g (54,000 rpm) at 37°C for 1 hr. Afterward, the pellet and supernatant were separated and placed on ice. The pellet was resuspended using 10 μM cytochalasin D in Mili-Q water. Both the dissolved pellet and supernatant were diluted 10-fold with Mili-Q water, combined with the appropriate amount of SDS sample buffer, and heated at 95°C for 2 min. Samples were separated using a 12% SDS PAGE gel and were then electrophoretically transferred onto a PVDF membrane. The blot was then probed with a 1:500 dilution of a proprietary antibody against actin for 1 hour at room temperature followed by incubation with an HRP-labeled anti-rabbit secondary at a dilution of 1:10,000 for 1 hour at room temperature. G and F actin signals were visualized using the ECL Plus detection system and were quantified from scanned blots using ImageJ.

### Statistics

Grubb's outlier test for continuous data was used to statistically determine the existence of outliers in the data. The Student's t-test was used to determine significant difference in the data, with a p-value less than 0.05 considered significant.

## Results

In order to evaluate the changes that a cell undergoes when exposed to ECM microtopography, less tumorogenic (Caco-2 E) and more tumorigenic (SW620) colon cancer cells were plated at subconfluent densities on PDMS scaffolds with varying microtopographies (Figure 1). PDMS scaffolds containing "pits" were used to emulate the microtopography found under well-differentiated tumor cells. PDMS scaffolds containing "posts" were created to emulate the microtopography found under poorly differentiated tumor cells. Planar PDMS scaffolds were used as a control. Caco-2 E cells are a less tumorigenic colon cancer cell-line derived from a tumor of unknown differentiation, which have a villous appearance and well-organized tight junctions on Transmission Electron Microscopy (TEM). In contrast, SW620 cells are a highly aggressive colon cancer cell-line originally derived from a lymph node metastasis [30] and have been shown to metastasize in nude mice [31]. Examples of each cell line on the three microtopographies we studied are shown in Figure 2.

### Impact of Microtopography on Rho A Activity

**Figure 3:**
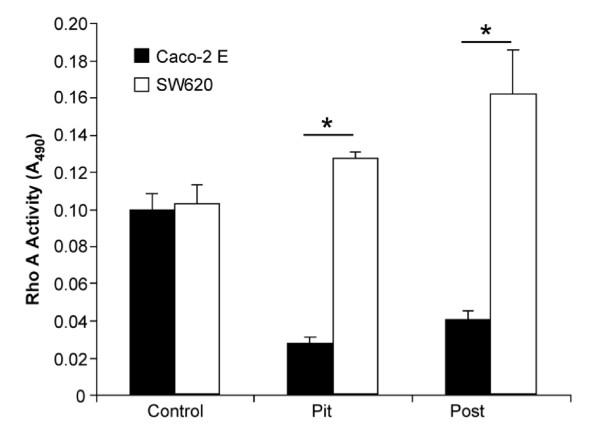
**ECM microtopography modulates Rho A activity in less and more tumorigenic colon cancer cells**. When plated on either a pit or a post microtopography, Caco-2 E cells exhibited a significant decrease in Rho A activity as compared to control (p < 0.01). In contrast, SW620 cells exhibited an increase in activity as compared to control when plated on either a pit or a post microtopography. In both Caco-2 E and SW620 cells, Rho A activity was higher on a post microtopography than on a pit microtopography. When plated on either a pit or a post microtopography, the Rho A activity of SW620 cells was significantly higher than that of Caco-2 E cells (* = p < 0.01). Rho A activity was determined using a colorimetric Rho A activity assay from Cytoskeleton, Inc.

As shown in Figure 3, Rho A activity levels were similar when either cell-line was plated on a planar surface. When Caco-2 E cells were plated on either a pit or post microtopography, the Rho A activity observed decreased significantly as compared to the control (p < 0.01). When SW620 cells were plated on a pit microtopography, the Rho A activity observed was similar to the level seen in the control. However, when SW620 cells were plated on a post microtopography, there was a nearly 50% increase in Rho A activity as compared to the control. Overall, the Rho A activity observed in Caco-2 E cells was lower than the activity observed in SW620 cells plated on either a pit or a post microtopography.

### ECM Microtopography Driven Changes in Motility are Rho A Dependent

While the above data suggests that changes in microtopography lead to alterations in Rho A activity in particular cell types, it does not fully explain how the microtopography of the ECM affects the Rho A activity. Furthermore, in the case of Caco-2 E cells, it may be that Rho A activity is not even important as it is reduced nearly five-fold over control. In order to answer the questions raised by this data, Rho A dependent motility was evaluated using time-lapse photography. Cell motility data was subsequently analyzed for degree of directed diffusion and confined diffusion using mean square displacement (MSD)(See Methods).

This method was employed because individual cells do not always move along a simple path (i.e. - a straight line), but instead cells respond to mechanical interactions, such as collisions with other cells or topographical barriers--and chemical signals which cause the cell to change direction and display random walk behavior, in which each step taken by the cell is in a random direction from the previous step. As a result of this random walk behavior, cellular motility cannot be accurately defined by linear velocity as both distance and directionality must be taken into account. Mean square displacement (MSD) evaluates motility by taking the sum of the distance squared; therefore each value in the summation is positive and grows larger with each time step. Furthermore, MSD is able further characterize cellular motility outside of cell speed by identifying diffusion parameters as well as cell movement patterns.

When MSD is plotted against time, a straight line is indicative of simple diffusion of the cell (Equation 1). A positive curve indicates directed diffusion or a systematic motion, which becomes more dominant as time increases (Equation 2). A negative curve indicates that the cell has confined diffusion or is moving within a confined space or cage in which the cell interacts with barriers that retard cellular motility. The linear portion of the curve can be modeled using Equation 3[32].(1)(2)(3)

D refers to the diffusion coefficient and is in μm^2^/min. *t *refers to the time interval in minutes. D_M _(μm^2^/min) refers to the diffusion coefficient of confined cells when not confronted by a barrier. Λ is the distance between barriers in μm, where the barriers can be from the microtopographical features of the scaffolds or from the presence of other cells. *v *is the velocity in μm/min.

**Figure 4:**
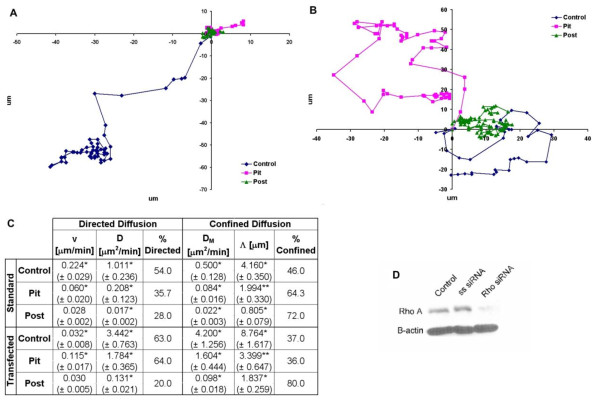
**ECM microtopography retards motility of less tumorigenic colon cancer cells via Rho A**. Microtopography has a significant inhibitory impact on the motility of the less tumorigenic Caco-2 E cells as can be seen by the decrease in both the cellular velocity and the movement coefficients when plated on either a pit or a post microtopography (panel A and panel C, p < 0.01). The overall result is a decrease in the total distance traveled by the Caco-2 E cells when plated on either a pit or a post microtopography (panel A). When Caco-2 E cells transfected with Rho A siRNA are plated on pit microtopography--which emulates the native environment of less tumorigenic cells--there is an increase in cell motility suggesting that Rho A plays an integral role in regulating cell motility in less tumorigenic cells (panels B and C). Panel D is a representative western blot showing successful knockdown of Rho A. The x and y axes in panels A and B are in μm and the colored lines are representative cell trajectories. The values in panel C are the mean ± the standard error. Where: * = p < 0.01, ** = p < 0.05 when comparing the standard and transfected values.

Using MSD, we observed the motility of both Caco-2 E and SW620 cells without and with Rho A siRNA. Effective knockdown of Rho A siRNA is demonstrated in Figure 4 panel D. Scrambled siRNA sequences were used as controls and we observed a more than 80% reduction in Rho A expression.

MSD analysis of the motility of Caco-2 E cells plated on a planar surface, (Figure 4) demonstrated almost equal distribution of cells that displayed directed and confined diffusion. Analysis of the motility of this cell type on either a pit or a post microtopography revealed that the number of Caco-2 E cells displaying directed diffusion had decreased suggesting that the pits and posts on these surfaces were acting as barriers to cell movement. Not surprisingly, we also observed that cellular velocity and average distance traveled by Caco-2 E cells decreased by 73% on a pit microtopography and by 87% on a post microtopography as compared to cells plated on a planar surface.

Our analysis of Rho A activity in Caco-2 E cells plated on either a pit or a post microtopography revealed a significant decrease in Rho A activity (Figure 3) suggesting that Rho A might not be involved in Caco-2 E response to topography. This was, however, not the case and knockdown of Rho A resulted in a significant increase in both directed and confined movement in Caco-2 E cells pated on either a planar surface or a pit microtopography (p < 0.01)(Figure 4, panel B and C). In other words, these cells lost their response to topographical cues and migrated erratically. As a result, there was an increase in the overall distance traveled by transfected cells on a pit microtopography as compared to the distance traveled by untransfected cells on the same microtopography (Figure 4, panel C). But, there was virtually no impact to the cellular velocity of cells plated on a post microtopography. As such, we conclude that despite the low level of Rho A activity in Caco-2 E cells plated on either pits or posts, this protein plays a role in maintaining cell confinement. This is a good thing in the setting of colon cancer.

**Figure 5:**
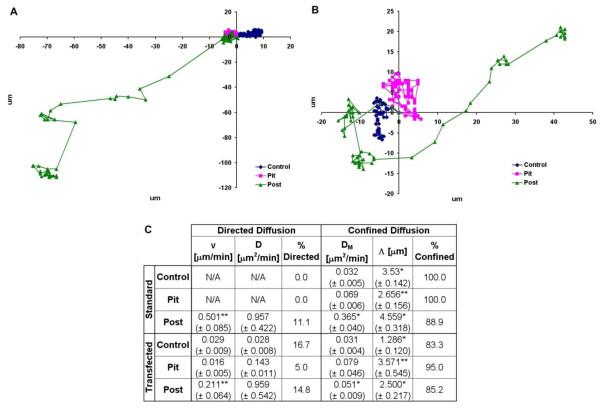
**Rho A induced by the ECM microtopography promotes motility of more tumorigenic colon cancer cells via Rho A**. Microtopography plays an integral role in the up regulation of cellular motility of the more tumorigenic SW620 cells when plated on a post microtopography (panel A and C). The result is an increase in the overall distance traveled by SW620 cells when plated on a post microtopography (panel A). When SW620 cells are transfected with Rho A siRNA, cellular motility was shown to decrease on the post microtopography (panel B and C). Since the post microtopography emulates the native environment of more tumorigenic cells, this data suggests that Rho A promotes the cellular motility of more tumorigenic cells in their native topographical environment. Proof of our ability to successfully knockdown Rho A using siRNA is shown in Figure 4, panel D. Similar results were obtained using SW620 cells (Data not shown.). The x and y-axes in panels A and B are in μm and the colored lines are representative cell trajectories. The values in panel C are the mean ± the standard error. Where: * = p < 0.01, ** = p < 0.05 when comparing the standard and transfected values.

In contrast to Caco-2 E cells, Rho A activity increased in SW620 cells plated on either a pit or post microtopography. But, when their motility was observed, they exhibited extremely confined movement and were relatively immotile when plated on a planar surface or a pit microtopography (Figure 5). Intriguingly, when plated on the post microtopography--which emulates the native environment of the tumorigenic SW620 cell-line--the motility increased significantly as shown in the cellular trajectory of Figure 5, panel A (p < 0.01). Furthermore, 11% of these cells exhibited directed movement. Overall, despite the barriers imposed on them by the post microtopography, SW620 cells traveled a greater distance from their originating point to their ending point. This is a significant finding when we compare the response of this highly tumorigenic cell line to that of less tumorogenic Caco-2 E cells, which were rendered immotile by the barriers of the post microtopography.

The impact of Rho A knockdown on SW620 cells, resulted in very little change in the percentage of confined cells on any surface when compared to that of the untransfected cells (Figure 5, panel C). However, on the post microtopography, the cellular velocity decreased by over 50% and the confined movement coefficient decreased by 7-fold. In short, both the motility and cellular confinement of SW620 cells decreased when plated on post microtopography in the absence of Rho A, suggesting that Rho A plays an integral role in motility of these more tumorigenic cells.

Taken together, this data suggests that topography-dependent changes in cellular confinement and motility are mediated by Rho A. It also demonstrates that a tumorigenic cell (SW620) has the ability to overcome barriers that would normally retard cell behavior, which suggests that these barriers may actually promote the motility of tumorigenic cells.

### ECM Microtopography Induces Changes in the Actin Cytoskeleton

Our motility data demonstrates that Caco-2 E cells were most motile and had the highest Rho A activity on a planar surface (control) and that tumorigenic, SW620 cells were most motile and had the highest Rho A activity on a post microtopography. Furthermore SW620 cells were more sensitive to Rho A knockdown suggesting that the role of Rho A in this tumorigenic cell-line is to overcome the mechanical barriers of the ECM.

**Figure 6:**
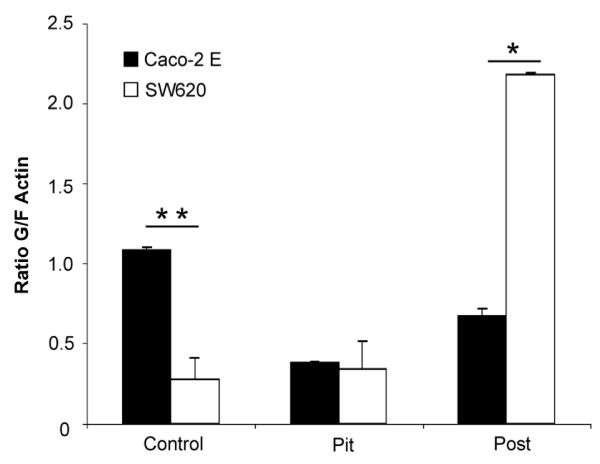
**The Microtopography of the ECM significantly impacts G/F actin ratios in less and more tumorigenic colon cancer cell-lines**. The G/F actin ratios in Caco-2 E cells plated on either a pit or a post microtopography was significantly lower as compared to cells plated on a planar surface (p < 0.05). In contrast, the G/F actin ratio in SW620 cells plated on a post microtopography was significantly higher than as compared to cells plated on either a planar surface or a pit microtopography (p < 0.01). When plated on a planar surface, the G/F actin ratio of SW620 cells is significantly lower than the G/F actin ratio of Caco-2 E cells. Where as, the G/F actin ratio in Caco-2 E cells is significantly lower than SW620 cells when plated on a post microtopography. (* = p < 0.01; ** = p < 0.05).

Since Rho A is a critical mediator of actin cytoskeletal turnover, we next evaluated the response of the actin cytoskeleton in both cell lines to each microtopography. To accomplish this, we determined the ratio of globular (G) to filamentous (F) actin (Figure 6).

In less tumorogenic, Caco-2 E cells the ratio of G/F actin in was significantly lower when they were plated on a pit or post microtopography as compared to the control surface (Figure 6, black) (p < 0.05). This finding supported the data shown in Figure 4, which showed that these cells were most motile on a control surface.

Also confirmatory of the motility data shown in Figure 5, the G/F actin ratio in tumorogenic, SW620 cells plated on either a control surface or a pit microtopography was low. However, the G/F actin ratio in SW620 cells plated on a post microtopography was significantly higher than the ratios of these cells plated on either a control surface or a pit microtopography (p < 0.01) suggesting that there is indeed significant actin turnover (Figure 6, white).

Overall, this data confirms our motility findings and lends further support to our supposition that the microtopography critically regulates cell behavior via Rho A signaling.

## Discussion

In this study, we have shown microtopography drives changes in cellular behavior in colon cancer cells via Rho A. Furthermore, we have demonstrated that the response of cancer cells to a particular microtopography is linked to their tumorogenicity. As such, this data is important because it not only identifies the microtopography of the ECM as an important biophysical regulator of colon cancer behavior but it also helps explain the seemingly disparate results regarding the role of Rho A in cancer and in particular, in colon cancer.

To date the best data regarding the role of Rho A in cancer comes from the epithelial to mesenchymal transition (EMT) literature. Specifically, cDNA array studies in melanoma have linked Rho A to EMT [33]. Rho A was also linked to EMT in a mouse model of melanoma [34]. In other epithelial cell models, Rho A has been linked to EMT through TGF beta signaling [35]. In contrast, when LIMI1863 colon cancer cells--a cell-line that forms spheroids in culture--were treated to induce EMT, Rho A expression was down-regulated [36]. Rho A has also been shown to be targeted for degradation by Smurf1 which in turn leads to a loss of tight junctions [37]. While our study is not specifically focused on EMT, it does address factors that are important to EMT; specifically, how topographical features of the ECM increase cell motility, a process crucial to the initiation of metastasis. Furthermore, while some work has been done looking at the contribution of mechanical signals, such as Rho A, to colon cancer behavior [38], none of this previous work has looked at the contribution of mechanical factors such as matrix topography.

However, the disparate responses of tumorogenic and non-tumorigenic colon cancer cells to varying microtopographical conditions indicates that one must consider both the tumorigenicity of the cell and the microtopography of the ECM when evaluating the role of Rho A in EMT and colon cancer. In other words, Rho A does indeed have the ability to both retard and promote EMT depending on both the cellular and mechanical features of the tumor.

## Competing interests

The authors declare that they have no competing interests.

## Authors' contributions

RR did the mathematical analysis of data and conducted some of the molecular biology experiments. JH, RV, MB, and CP also conducted some of the molecular biology experiments and helped create the original masks for the experiments performed herein. VM, RTST, and MC provided engineering insight into the project. SG wrote the manuscript and is the corresponding author on this paper.

All authors have read and approved the final manuscript.
